# Influence of good manufacturing practices on the shelf life of refrigerated fillets of tilapia (*Oreochromis niloticus*) packed in modified atmosphere and gamma-irradiated

**DOI:** 10.1002/fsn3.41

**Published:** 2013-05-30

**Authors:** Maria Lúcia Guerra Monteiro, Eliane Teixeira Mársico, Sérgio Borges Mano, Claudia Emília Teixeira, Anna Carolina Vilhena da Cruz Silva Canto, Helio de Carvalho Vital, Carlos Adam Conte-Júnior

**Affiliations:** 1 Programa de Pós-Graduação em Higiene Veterinária e Processamento Tecnológico de Produtos de Origem Animal, Faculdade de Veterinária – UFF, Rua Vital Brazil Filhon 64, 24.230-340, Santa Rosa, Niterói, Rio de Janeiro, Brazil; 2 Departamento de Tecnologia de Alimentos, Faculdade de Veterinária – UFF, Rua Vital Brazil Filhon 64, 24.230-340, Santa Rosa, Niterói, Rio de Janeiro, Brazil; 3 Centro Tecnológico do Exército – CTEx, Avenida das Américasn 28.705, 23020-470, Guaratiba, Rio de Janeiro, Brazil

**Keywords:** Conservation methods, irradiation, modified atmosphere packaging, tilapia

## Abstract

This study evaluated the influence of good manufacturing practices (GMP) on the shelf life of refrigerated fillets of Nile tilapia (*Oreochromis niloticus*) packed in modified atmosphere packaging (MAP) and irradiated. In a first series of experiments, 120 tilapia fillets kept under controlled sanitary conditions were purchased from a fish market managed by a cooperative. A second lot totaling 200 tilapia fillets was obtained under controlled storage conditions from a pilot plant. The combined effects of MAP (40% CO_2_ and 60% N_2_) and irradiation (1.5 kGy) were investigated by monitoring physical and chemical (total volatile bases and pH), bacteriological (aerobic heterotrophic mesophilic and psychrophilic bacteria) and sensory (acceptance test) changes in the samples. The quality of samples decreased with storage time regardless of the treatment, remaining higher in fillets produced in the pilot plant in comparison with the commercially produced fillets. The observed shelf life of nonirradiated commercially produced fillets was only 3 days, compared to 8 days for those produced in the pilot plant, probably due to GMP in the latter. It was concluded that, even with a combination of proven conservation methods for meats, the adoption of good manufacturing practices still remains essential before, during, and after the filleting process in order to ensure the effectiveness of the entire treatment.

## Introduction

Tilapia (*Oreochromis niloticus*) is one of the most important species in aquaculture because of its genetic, reproductive, and marketing characteristics. Although these fish can be raised under controlled conditions, their flesh is a complex biochemical structure that is easily metabolized by microorganisms and is rapidly affected by autolytic enzymes, due to its high water content, near-neutral pH, and predominance of unsaturated fat (Mársico et al. 2006). Therefore, investigations of alternative methods that could enhance the safety of this important food by preserving its physical, chemical, microbiological, and sensory attributes over longer storage times without significantly altering its nutritional content are desirable.

Modified atmosphere packaging (MAP) has the potential to retard microbial growth, extending the shelf life of foodstuffs (Mano et al. 2000). Irradiation is highly effective in reducing the initial microbial load, and often prolongs the shelf life of food without significantly changing its sensory characteristics and nutritional value (Vital and Freire 2008).

Consumers are becoming increasingly concerned with issues related to the nutritional, microbiological, sensory, physical, and chemical quality of fresh foods. However, despite the significant effects of MAP and irradiation on increasing the shelf life of products, the two methods do not reverse food spoilage and do not produce persistent effects. This makes it necessary to adopt good manufacturing practices (GMP) in all stages of processing in order to ensure effective treatment and hygienic quality of food (Lopes et al. 2004).

Although MAP and irradiation have been extensively studied in order to develop proper procedures for extending the shelf life of tilapia, to our knowledge, no studies have examined the influence of manufacturing practices on the technologies used to increase the shelf life of this fish species.

The goal of this study was to evaluate the effects of GMP on the shelf life of refrigerated fillets of Nile tilapia (*O. niloticus*) treated with irradiation and packed in a modified atmosphere.

## Materials and Methods

### Fish samples

In the first step, 120 (25 kg) fresh tilapia fillets were obtained from a commercial fish processing firm in Rio de Janeiro, during its routine functioning, under normal sanitary conditions established by the firm. In the second step, 200 (45 kg) fresh tilapia fillets were obtained from a pilot plant in Rio de Janeiro, which was designed to minimize the operational deficiencies that usually occur in commercial fish processing. The filleting process in the pilot plant was performed according to GMP established by the International Standards Organization – ISO (ISO – International Standards Organization 2005). The mean weight was 619 (±52) and 622 (±49) g for whole fish and 128 (±9) and 132 (±11) g for filleted fish that were commercially produced and produced in the pilot plant, respectively. The mean weights of the two sets of samples did not differ significantly. Both sets of samples were obtained in the same period (August) in order to avoid any effects of seasonal variations such as ambient temperature, humidity, and precipitation.

The samples were divided into eight lots according to treatment and origin. The commercially produced samples were labeled: T1 (control sample), T2 (MAP – 40/60 co_2_/N_2_), T3 (gamma radiation – 1.5 kGy), and T4 (MAP and gamma radiation, using the same conditions as treatments 2 and 3). The samples produced in the pilot plant were: T5 (control sample), T6 (MAP – 40/60 CO_2_/N_2_), T7 (gamma radiation – 1.5 kGy), and T8 (MAP and gamma radiation, using the same conditions as treatments 6 and 7). The samples were transported in isothermal containers with ice (0 ± 1°C) to the laboratory, and the physical, chemical, microbiological, and sensory analyses were performed periodically.

### MAP and gamma radiation process

The MAP was performed under laboratory conditions. Each fillet was packaged individually in a Cryovac BB4L bag, in a FAMABRAS (Model TEC MAQ AP 450; Itaquaquecetuba, São Paulo, Brazil) packaging machine (Conte Junior et al. 2010). The irradiation used a cesium-137 source, in the Centro Tecnológico do Exército (CTEx), located in Guaratiba, Rio de Janeiro. The samples, packed in plastic bags, were placed in the drawers of the irradiator (80 L capacity). Dry ice was placed in the drawers to prevent any excessive temperature increase during the irradiation process. The source activity of the radiation equipment was 45 KCi, where 0.9 kGy/h was used as the maximum dose rate. A computer program was used to calculate the effective doses and irradiation time, taking into account the density, size, and date of the samples. The samples for each treatment were irradiated for 1 h and 40 min.

### Physical and chemical analyses

The total volatile bases (mg TVB-N/100 g of sample) were determined according to the procedure of Conway, as stated in the manual of Siang and Kim (1992).

### Microbiological analysis

Microbiological methods were used to analyze the mesophilic and psychrophilic bacteria content, as recommended by the American Public Health Association (APHA – American Public Health Association 2001).

### Consumer sensory testing

Sensory evaluation was performed by the acceptance test with a 9-point hedonic scale, scored from 9 (strongly like) to 1 (strongly dislike), for a group of 30 untrained panelists (Stone and Sidel 1998). The attributes evaluated were color and general appearance. A score of 5 (neither liked nor disliked) was considered as the indifference region of the affective relationship of the judge to the product. Scores from 6 to 9 were considered as the acceptance region, and scores from 1 to 4 as the rejection region (Pereira et al. 2010).

### Statistical analysis

The results of the physical and chemical tests were analyzed using a second-degree polynomial regression. The linear regression with the Baranyi equation was applied to the parameters for bacterial development (latency phase and doubling time) of the curves obtained for the bacterial counts (Baranyi and Roberts 1994). In the sensory evaluation, the means and standard deviation associated with the judgments of the different attributes and treatments were calculated. An analysis of variance and Tukey test, at the 5% significance level, were then applied to these results, using the GraphPad Program InStat 3.0 (Motulsky 1998).

## Results and Discussion

### Microbiological analysis

Brazilian legislation does not specify the maximum allowed limits for mesophilic and psychrotrophic aerobic heterotrophic bacteria counts in fish products. In this experiment, we established 7.0 log CFU/g as the maximum limit, in accordance with data provided by the International Commission on Microbiological Specifications for Foods (1988), because the fillets were rejected by sensory inspection when the bacterial count of the samples exceeded this value.

**Figure 1:**
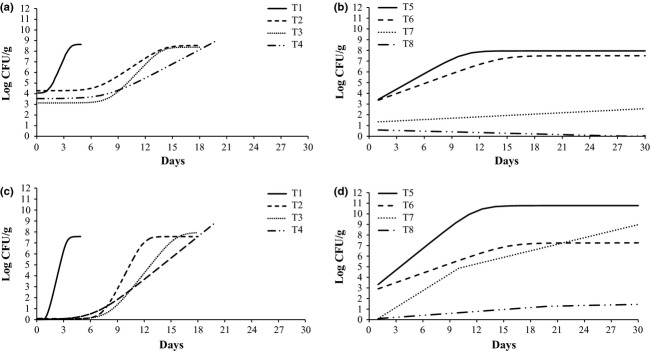
Linear regression of the mesophilic aerobic heterotrophic bacterial counts (a) and (b), and the psychrotrophic aerobic heterotrophic bacterial counts (c) and (d) of the tilapia fillets following treatments T1 (control sample), T2 (modified-atmosphere packaging – 40/60 co_2_/N_2_), T3 (gamma radiation – 1.5 kGy), T4 (modified-atmosphere packaging and gamma radiation, using the same conditions as treatments 2 and 3), samples from the fishery cooperative; and T5 (control sample), T6 (modified-atmosphere packaging – 40/60 co_2_/N_2_), T7 (gamma radiation – 1.5 kGy) and T8 (modified-atmosphere packaging and gamma radiation, using the same conditions as treatments 6 and 7), samples from the pilot plant.

The mesophilic and psychrophilic bacteria counts (Fig. 1) in the commercially produced fillets were performed after a shorter period of time (hours) because of the rapid growth of the bacterial population in these samples. After 3 h of storage, the bacterial growth in group T1 was 4.2 log CFU/g for mesophilic bacteria, without psychrotrophic growth. Mesophilic (5.5 log CFU/g) and psychrophilic (4.0 log CFU/g) bacterial growth started on the second day. After this storage period, the bacteria progressively multiplied, with a doubling time of 3.6 and 2.9 h, respectively. Before the third and fourth days of storage, the mesophilic and psychrophilic counts were 7.0 log CFU/g, respectively; that is, both counts reached the established limit.

The initial count of mesophilic and psychrophilic bacteria in the control samples produced in the pilot plant (T5) was 3.6 log CFU/g, and exceeded the limit (7.0 log CFU/g) only on the 12th and eighth days of storage, respectively. As seen in Figure 1, the initial count of the commercially produced samples was higher than in the samples produced in the pilot plant. Furthermore, the doubling time of mesophilic (3.6 h) and psychrophilic (2.9 h) bacteria of the commercially produced samples was lower than the doubling time of mesophilic (14.4 h) and psychrophilic (10.6 h) bacteria in the samples produced in the pilot plant. Therefore, the commercially produced samples showed significantly greater bacterial growth. This can be explained by the processing conditions in the plants where the samples were obtained. Under appropriate processing conditions, the shelf life of tilapia fillets can be increased by up to 4 days under refrigeration. This is 2.5 times longer than the storage period observed for the commercially produced samples.

The control samples (T1 and T5) showed higher bacterial counts than the samples from the treatments (2, 3, 4, 6, 7, and 8) during the entire storage period. This illustrates the beneficial effect of irradiation and MAP on extending the shelf life of tilapia fillets. However, the sanitary conditions in the processing plants where the samples were obtained may also have influenced the effectiveness of these treatments (Fig. 1).

The initial counts of the mesophilic and psychrophilic bacteria in the commercially produced samples with MAP (T2) were 4.2 and 2.9 log CFU/g, while the samples produced in the pilot plant showed 3.4 and 2.5 log CFU/g, respectively. Under controlled sanitary conditions, the bacterial counts were lower than in the commercially produced samples. MAP increased the doubling time of mesophilic and psychrophilic bacteria of the samples from both sites (pilot plant and commercially produced). During the storage period, the bacterial counts obtained in the commercially produced samples with MAP and the samples from the pilot plant (T2 and T6) remained stable, and were lower than the bacterial counts of the respective control samples (T1 and T5). For MAP, the bacterial doubling times were 12.5 h (mesophilic) and 13.7 h (psychrophilic) for the commercially produced samples (T2). These values were lower than the samples produced in the pilot plant (T6), which showed a 1-day doubling time for mesophilic and psychrophilic bacteria. Therefore, during the storage period, the samples produced in the pilot plant showed slower bacterial growth than in the commercially produced samples. This illustrates the importance of GMP, even when conservation methods are used. The mesophilic count of the commercially produced samples exceeded the limit on the 12th day, and the psychrophilic count exceeded the limit on the 15th day. The samples produced in the pilot plant reached the limit after the 15th and 17th days, but the mesophilic and psychrophilic count remained at around 7.2 and 7.5 log CFU/g, respectively, until the end of the experiment (30 days of storage under refrigeration). The MAP had a positive effect on reducing bacterial growth (Mano et al. 2000), which tripled the shelf life of the samples from both the commercial fish processing firm and the pilot plant. Soccol et al. (2005) found that MAP (40% CO_2_ and 60% N_2_) increased the shelf life of tilapia fillets. However, these authors observed lower bacterial counts (nearly 4.0 log CFU/g on the 20th day of refrigeration storage) compared to our study. This could be explained by the higher CO_2_ concentration and, consequently, the greater bacteriostatic effect of CO_2_ used by Soccol et al. (2005). The MAP effect was mainly observed in the samples produced in the pilot plant, which had a longer storage time than the commercially produced samples. The MAP apparently reduced the bacterial doubling time, especially in the samples produced in the pilot plant, which showed a lower initial count of microorganisms.

With respect to the irradiation method, the mesophilic counts were 2.2 and 1.8 log CFU/g in the commercially produced samples (T3) and the samples produced in the pilot plant (T7), respectively. In other words, the gamma radiation reduced the mesophilic bacteria count by 50%. The commercially produced samples exceeded the limit on the 12th day. The samples produced in the pilot plant did not reach 7.0 log CFU/g during the period of the experiment, because these samples were obtained from a plant with controlled sanitation conditions and showed lower initial counts of microorganisms (Fig. 1). The psychrophilic counts for treatments 3 and 7 reached the limit on the 15th and 23th storage days, respectively. The commercially produced samples showed an 8 h doubling time for mesophilic bacteria, and 11.3 h for psychrophilic bacteria. The bacterial doubling times (mesophilic and psychrophilic) of the samples produced in the pilot plant were 170.4 and 35.0 h, respectively. Irradiation damages the bacterial nucleic acids, reduces the initial count of microorganisms (Vital and Freire 2008; Monteiro et al. 2010), and increases the bacterial doubling time. Cozzo-Siqueira et al. (2003) found that 1.0, 2.2, and 5.0 kGy decreased the microbe content during storage when compared with nonirradiated samples. In agreement with our findings, these authors observed that the irradiated samples did not reach 7.0 log CFU/g until the 30th day of refrigerated storage.

The gamma radiation tripled the shelf life of the commercially produced tilapia fillets and the fillets produced in the pilot plant, suggesting the importance of GMP, even when conservation methods are used, because controlled sanitation conditions augmented the irradiation effect. According to Gunes et al. (2012), lower irradiation doses would likely be sufficient for commercial products, as the contamination levels would be lower than 5–6 log.

The mesophilic and psychrophilic bacteria counts for the samples treated with both MAP and irradiation (T4 and T8) were lower than the bacterial counts for the other treatments during the entire storage period.

The mesophilic and psychrophilic bacteria counts for the commercially produced samples (T4) exceeded the limit around the 16th storage day, and both showed a 15-h bacterial doubling time. The samples produced in the pilot plant (T8) did not reach the limit by the end of the experiment (30 days), and both counts indicated 15 days of bacterial doubling time (Fig. 1). Both the MAP and irradiated samples produced in the pilot plant and those commercially produced (T4 and T8) showed a longer bacterial doubling time than for the samples submitted to other treatments. This probably resulted from the combined effect of gamma radiation, which reduced the initial count of microrganisms, and MAP, which decreased the rate of bacterial growth. The dissolved CO_2_ lowers the pH, impeding the development, adaptation, and population growth of irradiation-resistant microorganisms (Monteiro et al. 2012). On the other hand, the mesophilic and psychrophilic bacterial doubling times of the commercially produced samples were lower than the bacterial doubling time of the samples produced in the pilot plant.

The MAP combined with gamma radiation increased the shelf life of tilapia fillets from the fishery cooperative and pilot plant by four and three times, respectively. Thus, the combined treatment was more effective than the individual treatments, possibly due to a combined effect of the irradiation and MAP. Gunes et al. (2012) observed that the use of MAP (50% CO_2_ and 50% N_2_) combined with irradiation (2 and 4 kGy) did not affect the radiation-induced inactivation of the pathogens, and inhibited the growth of the surviving pathogens and other microorganisms, increasing the shelf life of meatballs during refrigerated storage.

**Figure 2:**
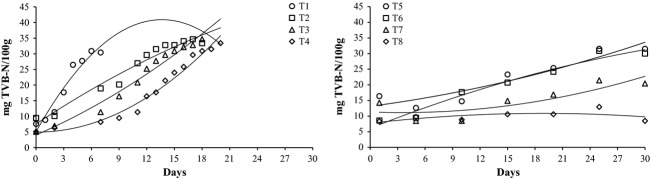
Second-degree polynomial regression of total volatile bases (TVB-N) of tilapia fillets following treatments T1 (control sample), T2 (modified-atmosphere packaging – 40/60 co_2_/N_2_), T3 (gamma radiation – 1.5 kGy), T4 (modified-atmosphere packaging and gamma radiation, using the same conditions as treatments 2 and 3), samples from the fishery cooperative; and T5 (control sample), T6 (modified-atmosphere packaging – 40/60 co_2_/N_2_), T7 (gamma radiation – 1.5 kGy) and T8 (modified-atmosphere packaging and gamma radiation, using the same conditions as treatments 6 and 7), samples from the pilot plant.

Independently of the treatment, the samples from the pilot plant were higher in quality than the commercially produced samples during the entire storage period (Figs. 1, 2), because samples produced in the pilot plant deteriorated more slowly due to the lower initial microbial load. This difference in microbial loads suggests that the pilot plant had better sanitation conditions.

The filleting process was considered the critical control point in an evaluation of the hazard analysis, and also the critical control point in the processing of tilapia fillets. To ensure the longest possible shelf life, it is essential to use preventive measures such as personnel training (Carbonera et al. 2006). In all the processing steps, the adoption of GMP is essential, because conservation methods such as MAP and irradiation cannot reverse the deterioration process (Satin 2002).

### Physical and chemical analyses

The TVB-N results consistently indicated an increasing deterioration of the fillets with storage time in all treatments, demonstrating a progressive reduction in the quality of the samples during the storage period (Fig. 2). However, the samples treated with irradiation, MAP, or a combination of both (2, 3, 4, 6, 7, and 8) deteriorated more slowly and had longer shelf lives in comparison with the control samples, demonstrating the positive effects of MAP, gamma radiation, and the two conservation methods combined.

Brazilian legislation (Ministry of Agriculture, Livestock and Supply 1997) does not define the allowed limits for the total volatile base (TVB) in freshwater fish. Therefore, it is necessary to correlate the physical, chemical, bacteriological, and sensory results to provide a basis for suggesting official limits for this food. In this study, the limit of 19 mg of TVB-N/100 g was adopted, as recommended by Soccol et al. (2005).

The TVB of the commercially produced control samples (T1) was 17.64 mg of TVB-N/100 g on the third storage day. The control samples produced in the pilot plant (T5) contained 13.86 mg of TVB-N/100 g on the eighth day under refrigeration. In this period, the sensory evaluation correlated with the other results of this experiment, and the commercially produced tilapia fillets showed undesirable sensory characteristics for consumption. The samples produced in the pilot plant gave better results than the commercially produced samples, because the samples were obtained under controlled sanitary conditions and had low initial counts of microorganisms.

The commercially produced samples with MAP, irradiation, and both conservation methods combined (T2, T3, and T4) showed 20.16, 20.79, and 21.42 mg TVB-N/100 g, exceeding the limit established in this study on the ninth, 11th, and 14th storage days, respectively. In this period, the bacteria count of the tilapia fillets exceeded the limit (7.0 log CFU/g), and the panel judges described a product with poor quality. In contrast, the samples produced in the pilot plant and submitted to the same treatments showed 20.70 mg of TVB-N/100 g (T6) and 21.42 mg of TVB-N/100 g (T7) on the 15th and 25th storage days, respectively. On the 30th storage day, samples produced in the pilot plant (T8) showed 8.44 mg of TVB-N/100 g. The bacterial counts for these samples did not reach the limit (7.0 log CFU/g), and the panel judges described no inappropriate sensory characteristics.

Cozzo-Siqueira et al. (2003) reported that doses of irradiation up to 5 kGy did not affect proteins (amino acids) and lipids (fatty acids) in tilapia muscle during 30 days of storage under refrigeration. However, MAP and irradiation can cause biochemical changes in tilapia fillets. The TVB showed a poor correlation with the other results of this study, and therefore was an inappropriate quality evaluation parameter for the refrigerated, modified atmosphere-packaged and irradiated fillets.

### Consumer sensory testing

Tables 1 and 2 show the consumer sensory scores of the commercially produced samples (T1, T2, T3, and T4) and the samples produced in the pilot plant (T5, T6, T7, and T8). The control samples (T1 and T5) showed mean scores that were intermediate between “strongly dislike” and “neither like or dislike,” on the fifth and 20th storage days for general appearance and color, respectively. This indicated that the judges perceived an indifferent sample quality, followed by a tendency to reject the product (Pereira et al. 2010). The commercially produced control samples (T1) deteriorated rapidly, which was reflected in the rapid development of undesirable sensory characteristics. The control samples produced in the pilot plant (T5) were judged acceptable for a period four times longer than the commercially produced control samples (T1).

**Table 1:** Consumer sensory scores of tilapia (*Oreochromis niloticus*) fillets commercially produced

	Attribute							
	
	Appearance				Color			
		
Days	T1	T2	T3	T4	T1	T2	T3	T4
0	5.90^a^	6.20^a^	7.00^a^	6.23^a^	5.96^a^	5.53^a^	6.76^a^	6.26^a^
2	6.06^a^	5.75^a^	6.46^a^	5.90^a^	5.73^a^	5.70^a^	6.30^a^	5.96^a^
5	2.63^b^	5.70^a^	6.33^a^	6.20^a^	2.66^b^	5.50^a^	6.23^a^	5.86^a^
8	ANP	6.00^a^	6.10^a^	5.00^a^	ANP	4.76^a^	6.06^a^	5.26^a^
12	ANP	4.73^a^	5.40^a^	5.33^a^	ANP	4.33^a^	5.13^a^	5.20^a^
15	ANP	3.33^b^	2.83^b^	5.10^a^	ANP	3.00^b^	2.33^b^	5.06^a^
19	ANP	ANP	ANP	4.16^b^	ANP	ANP	ANP	4.13^b^

T1 (control sample), T2 (MAP – 40/60 co_2_/N_2_), T3 (gamma radiation – 1.5 kGy), T4 (MAP and gamma radiation, using the same conditions of the treatment 2 and 3), samples produced in the pilot plant.

ANP: Analysis not performed, because the others parameters evaluated showed undesirable sensory characteristics of consumption.

Different letters in the same line indicate significant differences (*P* < 0.05).

**Table 2:** Consumer sensory scores of tilapia (*Oreochromis niloticus*) fillets produced in the pilot plant

	Attribute							
	
	Appearance				Color			
		
Days	T5	T6	T7	T8	T5	T6	T7	T8
1	6.76^a^	4.26^b^	5.73^a^	3.96^b^	6.53^a^	4.30^b^	5.66^a^	3.83^b^
10	6.63^a^	3.20^c^	4.90^b^	3.26^c^	6.73^a^	3.60^c^	4.96^b^	3.40^c^
20	5.63^a^	3.33^b^	5.20^a^	4.36^ab^	5.53^a^	3.43^c^	5.00^ab^	4.00^bc^

T5 (control sample), T6 (MAP − 40/60 co_2_/N_2_), T7 (gamma radiation – 1.5 kGy), T8 (MAP and gamma radiation, using the same conditions of the treatment 2 and 3), samples produced in the pilot plant.

Different letters in the same line indicate significant differences (*P* < 0.05).

On the first day, the treated samples (2, 3, and 4) received higher scores than the control samples (T1), but there was no significant difference (*P* > 0.05). The irradiated samples produced in the pilot plant (T7) received lower scores than the commercially produced control samples (T5), and again, the scores were not significantly different. Therefore, initially, irradiation did not affect the sensory quality of the tilapia fillets. In agreement with our findings, Ózden et al. (2007) reported that acceptability scores for texture and odor of sea bass were not affected by irradiation doses up to 5 kGy, but these scores decreased with longer periods of refrigerated storage. On the other hand, the modified atmosphere-packaged samples (T6) and the samples treated with both conservation methods (T8) differed significantly (*P* < 0.05) from the control samples (T5). This can be explained by the absence of oxygen and presence of CO_2_, which causes myoglobin deoxygenation and consequently discoloration of the tilapia fillets (Soccol and Oetterer 2003).

Modified atmosphere packaging slows bacterial multiplication (Mano et al. 2000) and gamma radiation reduces the initial count of microrganisms (Vital and Freire 2008). This delays the appearance of undesirable sensory changes and extends the shelf life of products. Therefore, the modified atmosphere-packaged and irradiated samples, and those treated with both conservation methods showed desirable quality characteristics for longer periods than the control samples. However, during the sensory analysis, the modified atmosphere-packaged samples showed “drip,” flaccid texture, and pale color, all critical attributes for the judges' evaluation. Meats packaged in a modified atmosphere with CO_2_ shows increased exudation, because the CO_2_ dissolved on the surface lowers the pH. Consequently, the water-retention capacity of the proteins decreases, which causes the increase in “drip,” flaccid texture, and pale color in the package (Soccol and Oetterer 2003). In addition, tilapia is considered a thin fish and has a large amount of water in its composition, which tends to produce exudation and affected the other attributes evaluated in this experiment (general appearance and color).

Modified atmosphere packaging combined with irradiation (T4) did not significantly influence (*P* > 0.05) the general appearance and color of the fillets by the 15th storage day, and maintained desirable sensory characteristics for a longer period. This suggests that the irradiation minimized the negative effects of MAP (dripping, flaccid texture, and pale color) (Monteiro et al. 2012).

## Conclusions

The control samples produced in the pilot plant maintained their quality over a longer storage period than the commercially produced control samples, demonstrating that GMP during the filleting process can increase the shelf life of refrigerated tilapia fillets. Furthermore, the conservation methods used (MAP, irradiation, and both methods combined) gave better results for the samples produced in the pilot plant than for the commercially produced samples during the entire storage period. Therefore, the adoption of GMP before, during, and after the filleting process is essential to obtain a product with a longer shelf life, even when using conservation methods that are effective, but have no persistent effect and do not reverse the deterioration process of foods.
